# Editorial: Lipids and Membrane Contacts in Yeast—Structure, Functional Aspects and Implications on Ageing, Cell Death and Autophagy

**DOI:** 10.3389/fcell.2022.881666

**Published:** 2022-04-12

**Authors:** Patrick Rockenfeller, Christopher T. Beh, Alexandre Toulmay

**Affiliations:** ^1^ Chair of Biochemistry and Molecular Medicine, Center for Biomedical Education and Research (ZBAF), University of Witten/Herdecke (UW/H), Witten, Germany; ^2^ Department of Molecular Biology and Biochemistry, Simon Fraser University, Burnaby, BC, Canada; ^3^ Centre for Cell Biology, Development, and Disease, Simon Fraser University, Burnaby, BC, Canada; ^4^ National Institute of Diabetes and Digestive and Kidney Diseases, National Institutes of Health, Bethesda, MD, United States

**Keywords:** membrane contact site (MCS), lipid droplet (LD), lipid trafficking and metabolism, autophagy, cell death

Membrane contact sites (MCSs) are junctions between different intracellular organelles that promote inter-membrane exchange and regulatory coordination. As sites enriched in lipid transfer proteins (LTPs) and small solute transporters, MCSs confer the “non-vesicular” direct exchange of lipids and small solutes (e.g., Ca^2+^) between closely apposed membranes, which act in parallel to canonical transport pathways for proteins and membrane components via vesicle trafficking (Phillips and Voeltz, 2016; Prinz et al., 2020). In fact, in the absence of vesicle transport some insoluble lipids such as ceramide, sterols, and specific phospholipids still move between cellular compartments unimpeded (Urbani and Simoni, 1990; Baumann et al., 2005; Hanada et al., 2009). In addition, some organelles, such as mitochondria and lipid droplets, are not even connected to the vesicular trafficking pathway suggesting that lipids move from and to these organelles only by non-vesicular means.

Although MCSs were originally identified almost 70 years ago (Bernhard et al., 1952; Bernhard and Rouiller, 1956; Porter and Palade, 1957), it has been within the last decade that many MCS proteins have been identified and their structural properties have been determined (Fernández-Busnadiego et al., 2015; Gallo et al., 2016; Reinisch and De Camilli, 2016; Hoffmann et al., 2019). Lagging behind this understanding, however, the functional roles of MCSs in the regulation of cell growth and physiology are still poorly defined.

Exploiting the molecular genetic tools available using *Saccharomyces cerevisiae*, recent discoveries detailing mechanisms of yeast MCS activities have focused on their regulatory significance in metabolic homeostasis (Henne et al., 2015; Jeong et al., 2017; González Montoro et al., 2018). MCS-dependent coordination of metabolism between specific membranes regulates organelle biogenesis, cellular quiescence, intracellular lipid and calcium mobilization/storage, and basic cellular bioenergetics (Kaufman and Malhotra, 2014; Herrera-Cruz and Simmen, 2017; Stefan et al., 2017; Farré et al., 2019). Given that many molecular components required for yeast MCSs share evolutionary conservation with homologues in higher eukaryotes, it is unsurprising that these studies in yeast provide valuable insights into MCS functions implicated in human disease (Rockenfeller and Gourlay, 2018; Prinz et al., 2020; Herker et al., 2021).

Still, many roadblocks remain in the MCS field, which need to be addressed in future investigations. This includes visualization of MCSs and proteins localized to MCSs, analysis of MCS formation dynamics, purification of organelle membranes and detection of lipid trafficking in live cells. The questions confronting current investigations into MCSs are: 1) How do the structural attributes of MCS proteins regulate lipid metabolism? 2) How does bulk lipid trafficking—in particular of phospholipids—work, and how is this regulated? 3) What growth conditions or genetic programs control the metabolic changes conferred by MCSs? And given the links between MCSs and disease pathology: 4) Do MCSs provide pharmacological targets for therapies combating the ever-increasing list of MCS-related disorders?

In this Research Topic we focus on functional aspects regulated through MCSs. These functional aspects impact inter-organelle communication, organelle structure, organelle inheritance and biogenesis, lipid and protein traffic, but also encompass cellular processes such as autophagy, membrane stress responses, cell death and ageing. Our knowledge about MCSs has exponentially increased throughout the past few years during which we have come to appreciate the importance of MCSs as platforms for general subcellular organization that dynamically coordinate multi-organelle processes. This research topic includes six review articles and one original research article that highlight recent advances in the field of MCS research, and describe the complex functional connections between lipid trafficking, metabolite channeling, autophagy, cell death and quiescence.

In the review article “A Unique Junctional Interface at Contact Sites Between the Endoplasmic Reticulum and Lipid Droplets” Choudhary and Schneiter summarize the current knowledge on ER-lipid droplet (LD) contacts, but also LD contacts with mitochondria and peroxisomes. In particular, this article discusses the function of specific LD-contact site components that generate the unique membrane interface that drives LD formation, lipogenesis and lipolysis. Emphasizing the cellular need to coordinate cellular lipid homeostasis between organelles, the authors also consider the roles of membrane LD-mitochondria and LD-peroxisome tethers, which support unique metabolic chemistries that are spatially segregated with cells. Because cellular bioenergetics and metabolism depends on LD interactions with these organelle membranes, the contribution of LD contacts on health and disease is also discussed.

Whereas Choudhary and Schneiter focus on LD biogenesis, Renne and Hariri concentrate on LD contacts with other organelles and their roles in fatty acid (FA) metabolism and trafficking. Their review article, “Lipid Droplet-Organelle Contact Sites as Hubs for Fatty Acid Metabolism, Trafficking, and Metabolic Channeling,” describe mechanisms by which LD contacts impact FA metabolism, including catabolism, synthesis, desaturation, elongation, and incorporation of FAs into complex lipids. In particular, the authors give insight into the importance of LD–ER contacts for metabolic channeling of FAs into LDs, which is an important requirement for lipid storage. They further discuss the function of LD contacts with peroxisomes and mitochondria for FA-beta-oxidation, and the roles of LD-vacuole contact sites for FA storage and mobilization.

In the review article “Mechanisms of Non-Vesicular Exchange of Lipids at Membrane Contact Sites: Of Shuttles, Tunnels and, Funnels,” Pascal Egea describes mechanistic features of lipid transfer proteins (LTPs) at the heart of MCSs. Based on structural and functional properties, LTP mechanisms are categorized into 1) diffusion, 2) sliding and 3) bridging-based. These mechanisms are discussed in depth as illustrated by the structural attributes of Ups1/Mdm35, ERMES, and other LTPs.

A particular focus on peroxisomal MCSs is presented in the mini-review “Peroxisomal Membrane Contact Sites in Yeasts” by Amit Joshi. Although these peroxisomal MCS include those with LDs as reviewed by Choudhary and Schneiter and Renne and Hariri, this article further extends to peroxisomal contacts with the ER, mitochondria, vacuole and plasma membrane. The author not only describes the structural features of these peroxisomal MCSs but also discusses functional aspects of peroxisomal MCSs important during processes such as peroxisome biogenesis.

Lenoir et al. also contribute to this special issue with their review article “Transport Pathways That Contribute to the Cellular Distribution of Phosphatidylserine”. The authors give a comprehensive overview of the current understanding on phosphatidylserine (PS) biosynthesis and the maintenance of its distribution in cellular membranes, including lateral distribution within bilayers. They discuss the intracellular trafficking of PS between membranes by different LTPs at ER contact sites with mitochondria, the plasma membrane, and with the autophagy isolation membrane. PS exchange between the two layers of the same membrane by flippases, and scramblases is also discussed.

The review article “Membrane-Interacting Antifungal Peptides” by Struyfs et al. takes a different viewpoint on lipid membranes. Here the authors focus on the membrane interaction of antimicrobial peptides (AMPs), in particular antimycotic peptides. Because fungal infections are on the rise worldwide, this article provides a fresh perspective on the growing knowledge on lipid membranes, as well as MCSs and their regulation, that can be exploited for medical and pharmacological purposes. In particular, the authors describe AMP structures and mechanisms allowing for specific fungal membrane interactions linked to programmed cell death, autophagy, mitochondrial dysfunction and MCSs.

In the original research article “Sterol metabolism differentially contributes to maintenance and exit of quiescence” Peselj et al. report on the role of lipids in the regulation of quiescence. They investigate the storage and consumption of neutral lipids upon glucose or phosphate starvation. The authors find that upon glucose exhaustion, LDs were degraded by lipophagy. Upon nitrogen exhaustion, however, cells exhibit a nucleus-vacuole junction expansion and accumulate LDs on the vacuole surface. Significantly, the formation of steryl esters and their hydrolysis and mobilization from LDs is critical for survival of phosphate starvation but not during glucose starvation. They conclude that the mechanism by which neutral lipid homeostasis adapts to both support quiescence and to exit from quiescence is specifically dependent on the limiting nutrient.

Altogether, this compilation delivers a focused picture of yeast MCSs and their impact on lipid homeostasis and other functional contributions to cell ageing, cell death, and autophagy.
